# Functional Mutations in the *VRTN* Gene Influence Growth Traits and Meat Quality in Hainan Black Goats

**DOI:** 10.3390/vetsci12100936

**Published:** 2025-09-26

**Authors:** Mengning Xu, Jing Huang, Ke Wang, Yuelang Zhang, Hanlin Zhou, Feng Wang, Jiancheng Han

**Affiliations:** 1 Zhanjiang Experimental Station, Chinese Academy of Tropical Agricultural Sciences, Zhanjiang 524013, China; Xmn07210955@163.com (M.X.); huangjing07182024@163.com (J.H.); lp_wangke@163.com (K.W.); zhouhanlin8@163.com (H.Z.); 2 Sanya Institute of Nanjing Agricultural University, Sanya 572024, China; caeet@njau.edu.cn; 3 College of Animal Science and Technology, Guangxi University, Nanning 530004, China; 4 Sanya Research Institute, Chinese Academy of Tropical Agricultural Sciences, Sanya 572024, China; 5 Hainan Institute of Zhejiang University, Sanya 572024, China; zhangyuelang@zju.edu.cn

**Keywords:** *VRTN* gene, SNP, Hainan black goat, growth traits, meat quality

## Abstract

The *VRTN* (vertebrin) gene plays a crucial role in regulating thoracic vertebral number in mammals; however, its function in goats remains largely unexplored. This study aimed to investigate genetic variations in the *VRTN* gene in Hainan black goats (HNBGs) and evaluate their associations with growth and meat traits. Using whole-genome and Sanger sequencing, we identified four SNPs in the *VRTN* gene, including three missense mutations (p.Pro615Ser, p.Arg490Lys, p.Thr476Met) and one synonymous mutation (p.Asp688Asp). Tissue expression profiling revealed high *VRTN* mRNA expression in skeletal muscle and low expression in internal organs, suggesting a potential role in muscle development. Temporal expression analysis indicated dynamic regulation during growth, with higher expression levels observed in early developmental stages. Association analyses revealed significant correlations between specific SNPs and key traits, including body length, chest circumference, carcass weight, and meat quality parameters. Notably, the p.Pro615Ser mutation was associated with a 0.441-fold reduction in *VRTN* expression and showed strong associations with multiple traits, underscoring its functional importance. These findings demonstrate that *VRTN* polymorphisms influence growth and muscle development in HNBGs, providing valuable insights for marker-assisted selection in goat breeding.

## Simple Summary

Hainan black goats are valued for their meat, but their slow growth limits productivity. We studied a gene called *VRTN*—known to affect spine development in mammals—to see if it influences goat growth and meat quality. We discovered four natural variations in this gene, three of which were linked to important traits like body size and meat tenderness. One variation (Pro615Ser) significantly reduced the gene’s activity and was associated with smaller body measurements and lighter carcasses. These findings help us understand how genetics shape goat growth and provide farmers with potential tools to breed better goats by selecting animals with desirable versions of the *VRTN* gene.

## 1. Introduction

The goat has been a valuable source of meat, milk, and fiber for humans since its domestication approximately 5000 years ago [1]. In modern agriculture, goat farming remains economically and culturally significant. Hainan black goats (HNBGs), an indigenous breed from tropical China, are known for their desirable meat quality, characterized by a chewy texture and high nutritional value [2]. However, this breed exhibits slower growth rates and smaller body size compared to northern Chinese breeds, limiting productivity [3]. Genetic improvement thus represents a promising approach to enhance their growth performance.

The *VRTN* (vertebrae development associated) gene plays a critical and multi-faceted role in regulating mammalian growth and development [4]. It is primarily known for its influence on vertebral patterning [5], where genetic variations, such as promoter insertions, directly increase thoracic vertebra count and extend body length, thereby providing a larger structural framework for muscle attachment and growth [6,7]. Beyond this well-established morphological mechanism, emerging evidence highlights its direct involvement in myogenesis and muscle regulation [8]. *VRTN* is hypothesized to modulate the expression of myogenic regulatory factors (e.g., MyoD, Myogenin) and may interact with key signaling pathways such as BMP and Wnt [9,10], thereby influencing both the hyperplasia and hypertrophy of muscle fibers. These pleiotropic effects—combining both skeletal elongation and direct molecular regulation of muscle development—make *VRTN* a highly valuable genetic target for livestock improvement. Selection for favorable *VRTN* alleles offers a promising strategy for enhancing overall body size, meat yield, and potentially meat quality, underscoring its potential application in marker-assisted breeding programs for productivity enhancement.

While most extensively studied in pigs, its role in goats remains poorly characterized. Emerging evidence suggests that *VRTN* may also influence muscle development and growth traits in caprines. In the former reseaches of our team, the sequencing result of *VRTN* shows it may have potential relationships with muscle development in goats [11]. This study aims to identify novel SNPs in the *VRTN* gene of HNBGs and evaluate their associations with growth and carcass traits. The findings may provide valuable genetic markers for breeding programs aimed at enhancing meat production in goats.

## 2. Materials and Methods

### 2.1. Animal Preparation and Ethics

The study utilized purebred Hainan black goats reared at the Danzhou Hainan Black Goat Breeding Conservation Farm located in northwestern Hainan Province, China. This provincial-level conservation farm specializes in the genetic preservation and propagation of this indigenous breed. All experimental goats were confirmed to have no crossbreeding within the last five generations, ensuring high genetic purity and breed authenticity. The animals were raised in a typical tropical environment characterized by high temperature and humidity throughout the year. All goats were maintained under standardized feeding and management conditions, receiving identical diets and veterinary care to minimize environmental variability. Housing conditions included well-ventilated barns with free access to water and shade to mitigate heat stress. Experimental procedures were reviewed and approved by the Institutional Animal Care and Use Committee of the Chinese Academy of Tropical Agricultural Sciences (Approval No. CATAS-2025006ZES). All sampling protocols strictly adhered to institutional ethical guidelines for animal research, with special attention to minimizing discomfort and stress during blood and tissue sample collection. Animal welfare was regularly monitored by trained veterinary staff throughout the study period.

### 2.2. Sample Collection and Phenotypic Trait Measurement

A total of 1200 blood samples were collected from two-year-old multiparous goats. Growth traits, including body height, chest depth, chest width, body length, chest circumference, withers height, hip width, body weight, and cross-section area of longissimus dorsi lumboismuscle were measured on these same individuals. Additionally, 617 goats of slaughter-appropriate age were selected for carcass trait evaluation. Measurements of water loss rate, shear force, carcass weight, cross-sectional area of the longissimus dorsi lumborum muscle, and water holding capacity (WHC) were conducted in accordance with the Chinese Agricultural Industry Standard [12] (NY/T 630-2002). For gene expression profiling, 11 tissues samples (heart, liver, brain, etc.) were collected from 12 goats. And longissimus dorsi lumborum muscle samples were obtained at six developmental time points (−30 days, 0 days, 0.5 years, 1 year, 2 years, and 3 years) from 18 goats for temporal expression analysis. Blood was stored at −20 °C, while other tissues and muscle samplzes were immediately snap-frozen in liquid nitrogen (−80 °C).

### 2.3. DNA/RNA Extraction and Primer Design

Genomic DNA was extracted from whole blood samples within 72 h post-collection using the Animal Blood Cells Genomic DNA Extraction Kit (Solarbio, Beijing, China), strictly following the manufacturer’s instructions. Total RNA was isolated from longissimus dorsimuscle tissue using TRIzol Reagent (Invitrogen, Carlsbad, CA, USA), treated with DNase I (Takara, Japan) to eliminate genomic DNA contamination. The quality and concentration of the extracted DNA and RNA were assessed using a NanoDrop™ 2000 Spectrophotometer (Thermo Fisher Scientific, Waltham, MA, USA), with all samples meeting the required purity criteria (A260/A280 ratios between 1.8 and 2.0). RNA integrity was confirmed by 1.0% agarose gel electrophoresis. To identify genetic variations in the *VRTN* gene, whole-genome sequencing data were aligned and compared against reference genomes available in the Ensembl database (release 110; accessed on 9 November 2024) and the Goat Genome Variation Database (GGVD; accessed on 9 November 2024). For genotyping and expression analysis, primers flanking four target SNP sites (chr1:17447378 G > A; chr1:17447632 G > A; chr1:17448006 C > T; chr2:17448048 G > A) were designed using the Primer-BLAST tool (NCBI, Table S1). Additionally, for qPCR amplification of *VRTN* transcripts, exon-specific primers were designed using the same platform, with *GAPDH* selected as the reference gene for normalization. All primers were subsequently validated for amplification efficiency and specificity under standard PCR conditions.

### 2.4. PCR/qPCR Amplification

PCR amplification was performed in 15 µL reactions containing 13 µL PCR master mix (TingSke, Shanghai, China) and 2 µL DNA template (10 ng/µL). Thermal cycling conditions followed Wang et al. [13], with an optimized annealing temperature of 60 °C. Amplicons were resolved by 1.0% agarose gel electrophoresis in 0.5× TBE buffer with nucleic acid stain (TingSke, Shanghai, China) and sequenced by Tsingke Biotechnology Co. (Beijing, China). SNP calling was conducted in BioXM 2.7.1 (NJAU, Nanjing, China) by aligning sequences to the reference genome (GenBank: NC_030817.1). qPCR analysis was performed using SYBR Green Master Mix (Vazyme, Nanjing, China) in 20 µL reaction volumes containing 0.4 µM each primer and 1 µL cDNA (equivalent to 50 ng RNA). Reactions were run in triplicate on a QuantStudio 6 Pro system (Applied Biosystems, Foster City, CA, USA) with the following protocol: 95 °C for 5 min; 40 cycles of 95 °C for 10 s, 59 °C for 30 s, 72 °C for 30 s; followed by melting curve analysis (65–95 °C with 0.5 °C increments) to confirm amplification specificity [14].

### 2.5. Statistical Analysis

Population genetic parameters and Hardy–Weinberg equilibrium (HWE) at the SNP loci of the goat *VRTN* gene were calculated using the SHEsis program (http://analysis.bio-x.cn, accessed on 24 February 2025) and the GENEPOP v4.7 (https://genepop.curtin.edu.au/, accessed on 24 February 2025). Protein structural impacts of missense mutations were predicted via SWISS-MODEL (https://swissmodel.expasy.org/, accessed on 15 June 2025). The association between SNPs in VRTN gene and growth traits in HNBGs was analyzed using a generalized linear model in the SPSS software (Version 18.0, IBM, New York, NY, USA), as follows:

Yhn=μ+Gh+en

where Y*hn* represents the phenotypic value, μ denotes the overall mean, G*h* corresponds to the fixed genotypic effect, and e*n* accounts for random residual error [13]. Relative *VRTN* expression levels were calculated using the 2^−ΔΔCt^ method, with normalization to the endogenous reference gene *GAPDH*, and the data derived from qPCR were analyzed using one-way analysis of variance followed by a post hoc test. All of the data were presented as the mean ± standard error (S.E.), and *p* < 0.05 was considered to be significant.

## 3. Results

### 3.1. Identification of Missense Mutations in the Goat VRTN Gene

**Figure 1:**
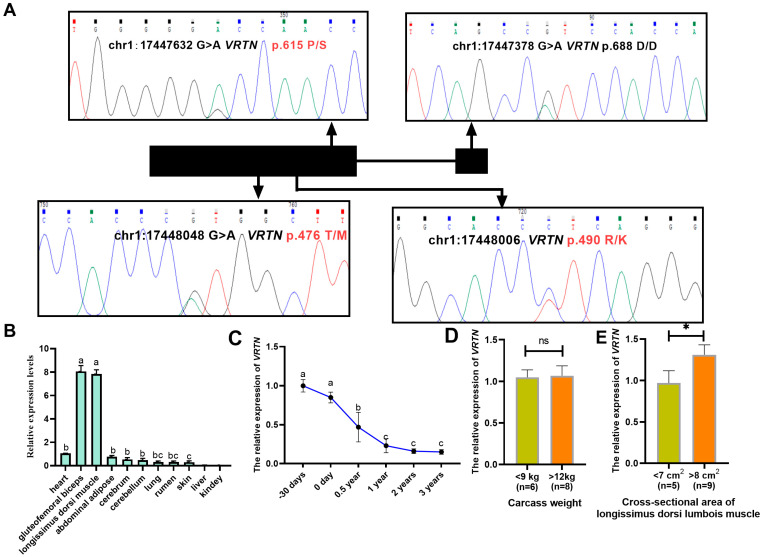
Genetic Variation and Spatiotemporal Expression Profiling of *VRTN* in Hainan Black Goats. (**A**) *VRTN* gene structure with SNP localization determined by Sanger sequencing. Exonic regions are denoted by black rectangles; missense mutations highlighted in red. (**B**) Tissue-specific *VRTN* mRNA abundance in adult females. Lowercase letters (a–c) denote statistically distinct expression patterns across tissues (*p* < 0.05). (**C**) Developmental regulation of *VRTN* transcription in longissimus dorsimuscle. Significant differential expression (*p* < 0.05) at distinct postnatal stages is indicated by letters (a–c). (**D**) *VRTN* expression correlation with carcass weight extremes. Asterisks (*) mark significant intergroup differences (*p* < 0.05). (**E**) Association between *VRTN* transcript levels and longissimus dorsi lumborumcross-sectional area variation. The * indicates significant differences (*p* < 0.05) between groups. All of the data were presented as the mean ± standard error (S.E.), and *p* < 0.05 was considered to be significant.

Using Sanger sequencing, we identified four SNPs in the *VRTN* gene: three missense mutations (chr1:17447632 G > A, p.Pro615Ser; chr1:17448006 C > T, p.Arg490Lys; chr1:17448048 G > A, p.Thr476Met) and one synonymous mutation (chr1:17447378 G > A, p.Asp688Asp). Mutation annotation was performed based on CDS alignment using the Ensembl and UniProt databases (accessed on 3 May 2025; Figure 1A). All four SNPs were originally planned for genotyping across the full cohort of 1195 individuals. However, only 300 samples were analyzed for SNP1 (p.Asp688Asp) due to its synonymous nature and limited functional relevance. Similarly, SNP4 (p.Thr476Met) was genotyped in only 300 animals owing to its extremely low minor allele frequency and absence of mutant homozygotes. Genotypic frequencies and population parameters are summarized in Table 1. Population genetic parameters revealed significant deviations from Hardy–Weinberg equilibrium for all SNPs (*p* < 0.05), likely stemming from artificial selection preferences within goat farming operations, and further exacerbated by the geographical isolation of Hainan Island. SNP2 (p.Pro615Ser) showing moderate polymorphism and heterozygote deficiency. Crucially, linkage disequilibrium analysis confirmed independent segregation of these variants (pairwise r^2^ < 0.1), suggesting distinct evolutionary trajectories (Figure S1).

### 3.2. Tissue-Specific and Developmental Expression of VRTN

Quantitative PCR analysis revealed that *VRTN* mRNA was highly expressed in skeletal muscles (specifically in the gluteofemoral biceps and longissimus dorsi), but was nearly undetectable in liver and kidney tissues (Figure 1B). Expression levels varied significantly across developmental stages: higher mRNA levels were observed during embryonic and early postnatal periods (30 days) compared to later stages (6 months), with a gradual decline during growth (Figure 1C). No significant difference in *VRTN* expression was detected between groups with different carcass weights (<9 kg vs. >12 kg; Figure 1D). Notably, individuals with a larger cross-sectional area of the longissimus dorsi lumborum muscle (>8 cm^2^) exhibited significantly higher *VRTN* expression than those with a smaller cross-sectional area (<7 cm^2^; *p* < 0.05; Figure 1E).

### 3.3. Structural Implications of Missense Mutations in VRTN

Protein structure prediction analysis revealed that the amino acid substitution corresponding to SNP3 (p.Arg490Lys) resulted in significant conformational alterations in the tertiary structure of VRTN. Localized structural divergences were observed in key functional domains (denoted by blue dashed circles in Figure S2), suggesting potential disruptions to hydrophobic core packing or hydrogen-bonding networks. These changes are predicted to adversely affect protein stability and may interfere with normal protein–protein interaction dynamics. In contrast, the other missense mutations did not yield substantial structural perturbations.

### 3.4. Association of VRTN Polymorphisms with Production Traits

Association analyses revealed significant correlations between VRTN polymorphisms and key production traits. For SNP2 (p.Pro615Ser), homozygous mutants exhibited significantly reduced chest width, chest circumference, hip width, carcass weight, and shear force compared to wild-type homozygotes. Similarly, SNP3 (p.Arg490Lys) mutants showed decreased chest circumference, body weight, carcass weight, water holding capacity, and shear force. Both SNPs demonstrated additive effects on multiple economically important traits, while non-significant associations are detailed in Table 2 and Table 3.

### 3.5. Effects of Genotype on VRTN Expression

**Figure 2:**
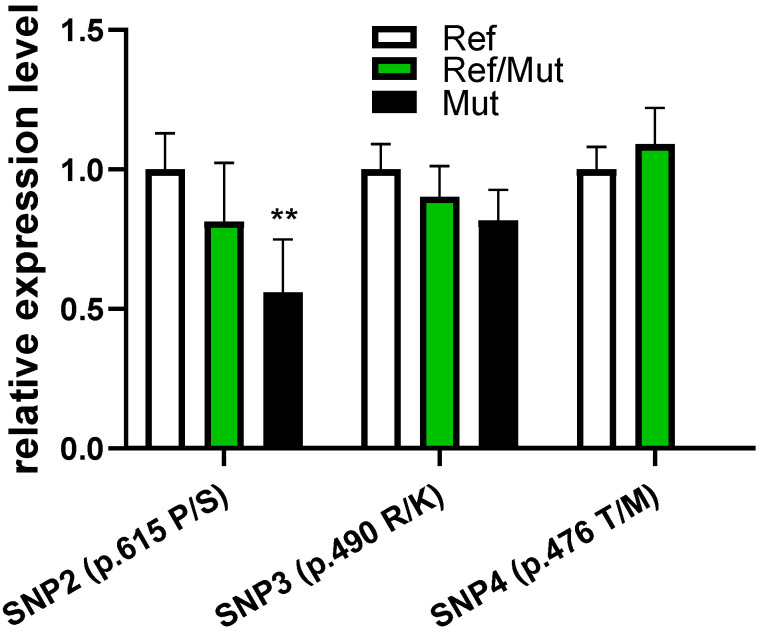
Missense mutations affect the expression of the *VRTN* gene in goat longissimus dorsi muscle. Ref, Reference genotype. Ref/Mut, Mutant heterozygous. Mut, Mutant homozygous. Comparison with normal genotype; ** indicates *p* < 0.01.

Genotype expression analysis demonstrated allele-specific transcriptional regulation (Figure 2). SNP2 (p.Pro615Ser) exhibited a striking dosage effect: homozygous mutants showed 55.9% expression reduction versus wild types, while heterozygotes displayed intermediate reduction. This suggests the mutation may disrupt transcription factor binding sites or mRNA stability elements. In contrast, SNP3 (p.Arg490Lys) caused no significant expression changes despite its strong phenotypic associations, implying protein-level functional impairment rather than transcriptional dysregulation. SNP4 (p.Thr476Met) heterozygotes showed minimal expression variation, consistent with its predicted neutral structural impact. The marked reduction in *VRTN* expression (*p* < 0.01) observed in SNP2 (p.Pro615Ser) homozygous mutants underscores its potential as a causal variant influencing muscle development.

## 4. Discussion

The vertebrate *VRTN* gene represents a pivotal regulator of axial development whose functional conservation across mammalian species underscores its fundamental role in musculoskeletal ontogeny [15]. Extensive research in porcine models has established that *VRTN* mutations modulate thoracic vertebral number through alterations in somite segmentation timing, thereby creating expanded skeletal templates for muscle attachment [16]. This indirect morphometric effect subsequently enhances longissimus dorsielongation and carcass yield [17]. Our preliminary findings suggest that similar mechanisms might operate in caprine species, where *VRTN* polymorphisms appear associated with economically important traits in Hainan black goats. Notably, our observations indicate that in addition to possible skeletal scaffolding effects, VRTN might participate in transcriptional regulation of myogenic pathways [18], a mechanism that could be particularly relevant in ruminants given their distinctive muscle fiber characteristics and metabolic adaptations [19].

We identified three missense mutations (p.Pro615Ser, p.Arg490Lys, p.Thr476Met) within conserved VRTN domains. Computational modeling predicts that p.Arg490Lys replaces a positively charged arginine with hydrophobic lysine at a putative protein–protein interaction interface (Figure S2), potentially impairing complex formation with developmental regulators like BMP2 or Wnt11 [9]. This structural perturbations may explain the observed functional consequences that molecular dynamics simulations suggest destabilization energies exceeding 2.5 kcal/mol, sufficient to alter folding kinetics and reduce functional half-life [20,21]. Such instability could compromise *VRTN* nuclear translocation efficiency, limiting its availability as a transcription factor for myogenesis-related genes (e.g., MYF5, MYOD [22,23]). During the initial population-level genotyping of all SNP loci, we genotyped 300 individuals for each locus. This analysis revealed that SNP4 exhibited an extremely low minor allele frequency and no homozygous mutant individuals were detected. Given its limited practical utility, we did not further investigate SNP4 in subsequent analyses. Similarly, SNP1, being a synonymous mutation, exhibits weak functional potential and thus was not prioritized as a primary focus of our study. Notbaly, all SNPs showed significant deviations from Hardy–Weinberg equilibrium, suggesting potential selective pressures or population stratification [24].

Tissue-specific profiling revealed *VRTN* mRNA abundance in HNBGs was more than 10-fold higher in skeletal muscles versus visceral organs (*p* < 0.01), with peak expression in longissimus dorsi, consistent with its role in axial musculature development [25]. Furthermore, temporal expression analysis demonstrated dynamic regulation during growth, in the early growth period (−30 days, 0 days), the high expression levels may align with the gene’s putative involvement, meaning the possible affection of *VRTN* gene in myogenesis procession. This trajectory parallels myoblast proliferation phases, suggesting *VRTN* facilitates satellite cell commitment during fetal myogenesis. Notably, individuals with larger longissimus dorsicross-sectional areas (>8 cm^2^) maintained 38% higher *VRTN* expression than counterparts (<7 cm^2^) even at maturity, implying persistent regulatory roles in physiological skeletal muscle hypertrophy [26], possibly through modulation of anabolic pathways like mTORC1 signaling [27].

Association analysis shows the relevance of these SNPs with traits. SNP2 (p.Pro615Ser) exhibited the most significant influence, markedly correlated with multiple growth and meat trait, and showing a marked reduction (0.441-fold) in mRNA expression in mutant homozygotes. This suggesting that the p.Pro615Ser may impair *VRTN* transcription or mRNA stability, ultimately impacting muscle-related traits [28]. Although p.Arg490Lys have weak effections on expression, it still associated with the key traits including body weight and carcass weight, means it may potentially regulate the growth procession of HNBGs. These variants exhibited no linkage disequilibrium, enabling independent selection for favorable alleles.

These findings suggest that the SNP2 (p.Pro615Ser) variant influences *VRTN* expression and may serve as a promising genetic marker for enhancing growth and muscle development in goats. However, further validation in larger populations is necessary to confirm its phenotypic effects. To fully understand its functional role, in vitro studies—such as *VRTN* knockdown/overexpression experiments in myocytes should be conducted to assess its impact on muscle differentiation [29]. Additionally, enzymatic activity assays of the mutant protein would help clarify its biochemical effects. Such investigations will strengthen its potential application in precision breeding for improved goat production.

## 5. Conclusions

We identified four SNPs in the *VRTN* gene of Hainan black goats, including three missense mutations associated with growth and meat quality traits. SNP2 (p.Pro615Ser) was correlated with altered *VRTN* expression, suggesting its potential functional role. These results propose *VRTN* as a candidate gene for muscle development, though further validation is required before breeding applications.

## Figures and Tables

**Table 1:** Genotypic frequencies and population parameters in the *VRTN* gene.

Loci	Size	Genotypic Frequencies			HWE	Population Parameters			
	*n*	Ref	Ref/Mut	Mut	*p*-Value	Ho	He	Ne	PIC
SNP1 p.Asp688Asp	300	110	179	11	*p* < 0.05	0.260	0.252	1.336	0.446
SNP2 p.Pro615Ser	1195	747	416	32	*p* < 0.05	0.304	0.282	1.392	0.321
SNP3 p.Arg490Lys	1195	972	202	21	*p* < 0.05	0.145	0.157	1.186	0.183
SNP4 p.Thr476Met	290	269	21	0	*p* < 0.05	0.093	0.088	1.097	0.088

Note: HWE, Hardy–Weinberg equilibrium. Ho, observed homozygosity. He, heterozygosity. Ne, effective allele numbers. PIC, polymorphism information content. Ref, Reference genotype. Ref/Mut, Mutant heterozygous. Mut, Mutant homozygous.

**Table 2:** The association analysis between the traits and SNP2 p.Pro615Ser in the goat *VRTN* gene.

Traits	Genotypes (Mean ± SE)			*p* Values
	Ref	Ref/Mut	Mut	
body height (cm)	52.52 ± 0.66	52.57 ± 0.73	52.47 ± 0.52	0.881
chest depth (cm)	26.23 ± 0.47	25.82 ± 0.74	26.12 ± 0.57	0.472
chest width (cm)	15.82 a ± 0.42	15.07 b ± 0.37	15.21 b ± 0.54	0.035
body length (cm)	66.23 ± 1.02	64.87 ± 0.83	65.33 ± 0.95	0.053
chest circumference (cm)	72.32 a ± 0.92	68.44 b ± 1.07	69.83 b ± 1.13	0.034
withers height (cm)	54.88 ± 0.82	54.08 ± 0.61	55.32 ± 0.97	0.363
hip width (cm)	18.53 a ± 0.86	16.96 b ± 0.69	17.82 a ± 0.60	0.043
body weight (kg)	27.31 ± 1.27	25.45 ± 0.72	26.35 ± 0.58	0.235
Carcass weight (kg)	9.78 a ± 0.41	9.34 b ± 0.31	9.29 b ± 0.37	0.039
cross-section area of longissimus dorsi lumboismuscle (cm^2^)	7.85 a ± 0.51	7.27 a ± 0.36	7.43 b ± 0.41	0.054
water loss rate (%)	4.62 ± 0.19	4.70 ± 0.21	4.55 ± 0.17	0.749
water holding capacity (%)	4.58 ± 0.23	4.63 ± 0.21	4.70 ± 0.34	0.613
shear force (N)	49.72 a ± 0.31	48.35 b ± 0.24	48.07 c ± 0.28	0.017

Note: letters (a, b) indicate significant differences (*p* < 0.05) between different genotypes and traits. Ref, Reference genotype. Ref/Mut, Mutant heterozygous. Mut, Mutant homozygous.

**Table 3:** The association analysis between the traits and SNP3 p.Arg490Lys in the goat *VRTN* gene.

Traits	Genotypes (Mean ± SE)			*p* Values
	Ref	Ref/Mut	Mut	
body height (cm)	53.42 ± 0.82	51.05 ± 1.47	52.08 ± 1.32	0.558
chest depth (cm)	26.31 ± 0.93	25.71 ± 1.27	25.44 ± 1.03	0.762
chest width (cm)	15.82 ± 0.71	14.99 ± 0.86	16.04 ± 1.20	0.33
body length (cm)	66.32 ± 2.07	64.98 ± 1.15	64.93 ± 1.72	0.702
chest circumference (cm)	73.81 a ± 1.24	69.93 b ± 0.91	70.30 b ± 1.29	0.042
withers height (cm)	55.62 ± 1.04	54.33 ± 0.90	54.85 ± 0.62	0.084
hip width (cm)	18.03 ± 1.02	17.36 ± 0.61	17.01 ± 0.72	0.062
body weight (kg)	27.48 a ± 0.67	27.35 a ± 0.46	26.42 b ± 0.41	0.028
Carcass weight (kg)	9.68 a ± 0.16	9.42 b ± 0.21	9.39 b ± 0.23	0.039
cross-section area of longissimus dorsi lumboismuscle (cm^2^)	7.54 ± 0.13	7.51 ± 0.10	7.49 ± 0.14	0.216
water loss rate (%)	4.63 ± 0.23	4.62 ± 0.08	4.78 ± 0.17	0.068
water holding capacity (%)	4.72 a ± 0.15	4.63 b ± 0.08	4.58 b ± 0.12	0.047
shear force (N)	49.03 a ± 0.41	47.97 b ± 0.28	47.38 b ± 0.14	0.008

Note: letters (a, b) indicate significant differences (*p* < 0.05) between different genotypes and traits. Ref, Reference genotype. Ref/Mut, Mutant heterozygous. Mut, Mutant homozygous.

## Data Availability

The original contributions presented in this study are included in the article/Supplementary Material. Further inquiries can be directed to the corresponding author.

## Supplementary Materials

The following supporting information can be downloaded at https://www.mdpi.com/article/10.3390/vetsci12100936/s1, Table S1: Primer information; Figure S1: LD Analysis of four SNPs in goat VRTN gene; Figure S2: Prediction of the protein structure change caused by the SNP3 p.490RK mutation; Supplementary Material S1: The differentially expressed genes and mutations identified through RNA-seq analysis of various quality muscles of HNBG.
