# Supplementary material for: Functional Mutations in the VRTN Gene Influence Growth Traits and Meat Quality in Hainan Black Goats
Source: Vet Sci. 2025 Sep 26;12(10):936. doi: 10.3390/vetsci12100936 (PMC12567611; doi:10.3390/vetsci12100936)
Supplement: Supplementary file 1 [file vetsci-12-00936-s001.zip › Tables S1.pdf]

Table S1. Primer information

Gene name		5'-3'	Length	Note
VRTN	F	GTCACTGTAGACACCGTGGG	951	chr10:17447256-17448206
	R	CTACTACAACTGGCGCCGAA		
	F	ATCATAGAGGGCGCGTTTCC	906	chr10:17447396-1744830117448301
	R	CGGGCCAAATTGTACCTGGA		
VRTN	F	GCTCCACCTATTACGCCTGG	181	mRNA-qPCR
	R	TCCAGGTACAATTTGGCCCG		
GADPH	F	TGAAGGTCGGTGTGAACGGATTTGG	277	mRNA-qPCR
	R	ACGACATACTCAGCACCAGCATCAC		
