# Supplementary figures and images for: Functional Mutations in the VRTN Gene Influence Growth Traits and Meat Quality in Hainan Black Goats
Source: Vet Sci. 2025 Sep 26;12(10):936. doi: 10.3390/vetsci12100936 (PMC12567611; doi:10.3390/vetsci12100936)

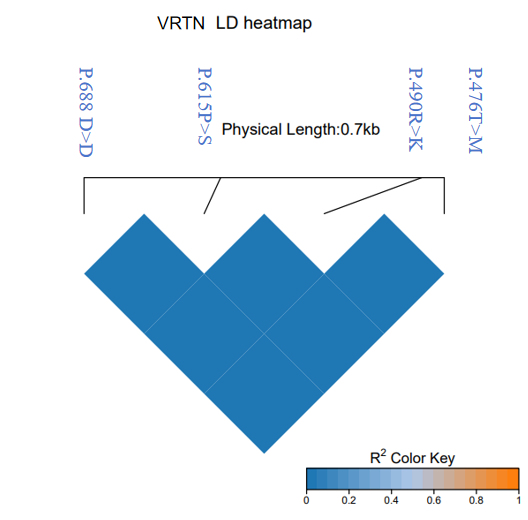

Supplement: Supplementary file 1 [file vetsci-12-00936-s001.zip › Figure S1LD Analysis of four SNPs in goat VRTN gene.png]

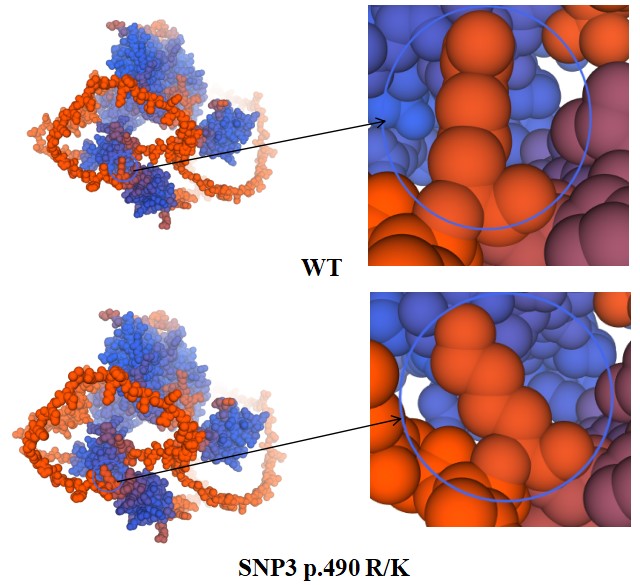

Supplement: Supplementary file 1 [file vetsci-12-00936-s001.zip › Figure S2 Prediction of the protein structure change caused by the SNP3 p.490RK mutation.jpg]
